# Revisiting Cattle Temperament in Beef Cow-Calf Systems: Insights from Farmers’ Perceptions about an Autochthonous Breed

**DOI:** 10.3390/ani11010082

**Published:** 2021-01-05

**Authors:** Laura X. Estévez-Moreno, Genaro C. Miranda-de la Lama, Morris Villarroel, Laura García, José Alfonso Abecia, Pilar Santolaria, Gustavo A. María

**Affiliations:** 1 Department of Animal Production & Food Science, Agri-Food Institute of Aragon IA2, University of Zaragoza, 50013 Zaragoza, Spain; genaro@unizar.es (G.C.M.-d.l.L.); levrino@unizar.es (G.A.M.); 2 CEIGRAM, ETSIAAB-Agricultural Production, Technical University of Madrid (UPM), 28040 Madrid, Spain; morris.villarroel@upm.es; 3 Aragonese Association of Farmers of Pyrenean Cattle Breed (ASAPI), 50194 Zaragoza, Spain; lauraasapi@gmail.com; 4 Department of Animal Production and Food Sciences, Environmental Sciences Institute (IUCA), University of Zaragoza, 50013 Zaragoza, Spain; alf@unizar.es; 5 Department of Animal Production and Food Sciences, Environmental Sciences Institute (IUCA), University of Zaragoza, 22071 Huesca, Spain; psantola@unizar.es

**Keywords:** cattle temperament, focus group discussion, human-animal interaction, farmers perception, autochthonous cattle breed

## Abstract

**Simple Summary:**

Temperament is a key issue in beef cattle production systems, as it defines the responses of animals to human handling and to potentially challenging or fear-eliciting situations. However, little is known about how farmers define or assess temperament. Focus group discussions were carried out in order to analyze the perceptions of farmers regarding a series of temperament traits of animals, focusing on the Pyrenean cattle breed. Farmers described animal temperament using several adjectives such as “strong”, “aggressive”, “nervous”, “fearful”, “brave”, or “smart”, which account for their integrative vision of this concept. They reported a differential long-term development of the temperament of cows and bulls and mentioned that external features are not reliable indicators of temperament as compared with animal behavior. Sensory acuity, such as sight, hearing, and smell, were thought to be related to temperament. The results of this study are useful to better understand the decisions of farmers about cattle buying, selling, or culling, and their perspective about temperament should be considered in the research and design of genetic improvement schemes.

**Abstract:**

Understanding temperament is an important part of cattle production since undesirable temperament may cause serious problems associated with aggression, maternal care, and human safety. However, little is known about how farmers define or assess temperament, especially in autochthonous cattle breeds. The aim of this study was to explore perceptions of farmers about the temperament of the Pyrenean cattle breed with special attention to beef cow-calf systems in Spain. The methodology used to obtain the information was focus group discussions (FGD). Farmers defined temperament as a behavioural response to challenging situations imposed by human handling. Specific terms used were related to active or passive reactions to fear (e.g., “strong”, “aggressive”, “nervous”, “fearful”). The speed of response to stimuli was also important. Female temperament was thought to become more docile with age while bull temperament was more variable. Maternal aggressiveness was highlighted as a potential human safety problem, but also desirable in an extensively bred animal who may need to defend calves against predators. Anatomical characteristics were seen as unreliable predictors of temperament, while behavioural indicators were more widely used, such as “alertness”, which was a general trait of the breed, and “gaze”, which, when associated with an alert expression, suggests a potential threat. Sensory acuity, such as sight and smell, were thought to be related with temperament in some FGDs but there was no overall agreement as to whether different behavioural responses were due to differences in sensory acuity. The results from the study could be useful during training programs or in the development of new genetic selection schemes and evaluation protocols involving cattle temperament.

## 1. Introduction

The study of individual differences in animal behavior that are consistent across contexts and across time is now wide-spread throughout animal sciences, giving rise to terms such as animal temperament, personality or coping styles [1,2,3]. Although some authors use these terms interchangeably, others like Finkemeier et al. [4] point out that temperament and coping styles overlap with personality, because they are sub-aspects of the whole personality concept. In farm animal research, the study of temperament has focused on analyzing behavioral responses of animals to potentially fear-eliciting or challenging situations related to production conditions, including responses to human handling [5]. Cattle temperament is important for the cattle industry since it is related to productive and reproductive performance, work safety, and animal welfare [2,3]. There has also been a trend towards the genetic selection of docile and safe-to-handle animals, which has further promoted research on cattle temperament, mostly based on the observation of specific parameters with different levels of participation by observers and the incorporation of precision measuring devices [6,7,8,9]. Widespread attention has been given to the relationship between cattle temperament and the response to specific (human) handling practices, heritability, and some productive parameters [10,11]. Meanwhile, human-centered research has focused on identifying certain management practices, attitudes, and other characteristics of stock people that may be linked to different facets of animal behavior and welfare [12,13,14].

However, there is still little research on cattle temperament from the farmers’ perspective. The progressive industrialization of beef and milk production and the concomitant increase in herd size may affect a farmer’s knowledge about individual cow temperament [15]. In spite of that, there are small and medium-scale farmers in different regions around the world that continue to handle cattle directly and are able to identify animals individually. This is the case for most farms with suckling cows of autochthonous breeds, used for meat production in Spain, which are usually managed by families in extensive or semi-extensive systems. Repeated handling and observation over months and years can provide this farmers with an integrated understanding of their animals’ behaviour and temperament [16]. Based on this premise, we used the Pyrenean breed to approach perceptions of Spanish farmers about cattle temperament, using focus group discussions (FGDs). The FGD methodology has been used before to explore farmers’ beliefs, perceptions, knowledge, and perspectives about several issues related to cattle production. These include calf health [17], welfare challenges and solutions [18], cattle dehorning [19], biosecurity [20], expectations and receptivity regarding animal welfare advice [21], and different topics related to epidemiology [22].

Pyrenean cattle are an autochthonous Spanish breed that was in danger of extinction during the 1960s and 1970s [23] but has recently increased in numbers, being now listed as a “Promoted Autochthonous Breed” by the Spanish Government, with its own herd book and genetic breeding scheme. Their conservation helps to support livelihoods and avoid rural depopulation in Spain, and their production systems, based on an extensive production system, meet multiple conditions to be commercialized under labels that incorporate sustainability criteria. From a scientific viewpoint, autochthonous breeds can be a useful source of information to understand the basis of bovine behavior, with possible applications in commercial breeds, because of their adaptation to diverse agroecosystem conditions and a shorter history of genetic specialization towards dairy or meat production. The aim of this study was to explore cattle farmer perceptions about the temperament of the Pyrenean cattle breed with special attention to cow-calf systems in Spain. Specifically, we wanted to describe commonly held views, as well as points of disagreement, regarding: (i) the main temperament-related traits of the breed, (ii) the ontogenetic and phylogenetic factors that may influence temperament, and (iii) the mechanisms through which farmers perceive animal temperament.

## 2. Materials and Methods

### 2.1. Study Description

The Pyrenean cattle breed is an autochthonous breed closely linked to the mountain landscapes of the Pyrenees. Although it has been described in works that go back to the 19th century, it was officially recognized as a breed in 1905, with the creation of the Herd Book, which has been maintained until today. Since 1920, it has been progressively specialized towards meat production, replacing the triple work-meat-milk aptitude that it previously had [24]. Although originally its population was concentrated in the Autonomous Communities of Navarra, the Basque Country and Aragon, it has spread to other regions of Spain, now being used on 1047 farms with a census of 26,399 cows (females > 2 years) currently registered in the breed’s Herd Book [25]. In general, cows feed on mountain pastures or grasslands (private or communal near their stables during the summer, spring and autumn). During the winter they are kept indoors with hay, cereal straw and sometimes given feed. The main differences in management are related to the characteristics of the grazing areas, the distance from the stables, and the places where animals spend the night. The calves are fattened in feedlots or on farms, generally using concentrate and straw. The study used focus group discussions (FGD) to collect data about participants’ knowledge and opinions regarding Pyrenean cattle temperament, using an inductive and exploratory approach. FGD is a qualitative research method where a small group of participants discusses a particular issue under the guidance of a moderator who keeps the discussion as focused, non-threatening and ‘natural-feeling’ as possible, with minimal self-involvement [26,27]. Four FGDs (Table 1) were held between March and June 2019, in the Autonomous Communities of Aragon (2), Navarra (1), and the Basque Country (1), which account for 67.9% of registered suckling cow population [25].

In each autonomous community, a virtual invitation to participate in the FGD was sent via the National Confederation of Pyrenean Cattle Breed Associations (known by its Spanish acronym CONASPI) to the members of the regional associations. In order to favor a certain compatibility and cohesiveness of the group and to facilitate the free expression of participants, some inclusion criteria were applied. Farmers needed to: (i) be currently employed as cattle owners (no retired farmers), (ii) have at least three years of experience in breeding Pyrenean cattle, (iii) be in charge of handling animals directly, and (iv) not be involved/aware of the scientific study of temperament. The farmers that finally attended the invitation were all male, between 25 and 65 years, from cattle farming families, having had direct contact with cattle of several breeds during most of their lifetime. The number of participants ranged from 8 to 11 people per FGD, an effective group size to encourage open discussion [28] with a total of 37 participants overall. A technician from the local farmer associations linked to CONASPI also attended each FGD, and was asked to act as an observer and limit input until the end of the discussion.

### 2.2. Interview Guide and Data Collection

A common meeting agenda was designed for each FGD to enable a joint analysis of all the material collected. Each FGD lasted between 60 and 90 min and was recorded (with participants’ consent) and facilitated by the same two researchers, a moderator and an assistant, neither having had previous contact with the participants. The moderator introduced each topic, promoted discussion, exhorted excessively talkative participants to let others talk, and encouraged all participants to speak, without introducing bias or pressures [28], while the assistant took notes on the content of the discussions and kept the time. At the beginning of the focus group, participants were informed about the objectives and dynamics of the activity, the anonymity and confidentiality, and the possibility to withdraw from the discussion at any moment. After recording verbal consent from all participants to be included in the study, they introduced themselves, mentioning their name and years of experience in Pyrenean cattle breeding. A semi-structured guide was developed with open-ended questions that were asked in all groups to collect farmer perceptions on the following three axes of discussion: (1) definition of the term “temperament”, characteristics of Pyrenean breed temperament and comparison with other known breeds; (2) anatomical and behavioural indicators of temperament and (3) ontogenetic and phylogenetic factors that may influence temperament. Some general questions were used to introduce and prompt the discussion of each topic (Table 2) in all FGDs, but, according to each FGD dynamic, additional follow-up questions were asked by the moderator when deemed necessary. At the end of each FGD, the moderator summarised the main topics covered (consensus and disagreements) and noted any unresolved issues for clarification from the group [29].

### 2.3. Data Analysis

**Figure 1:**
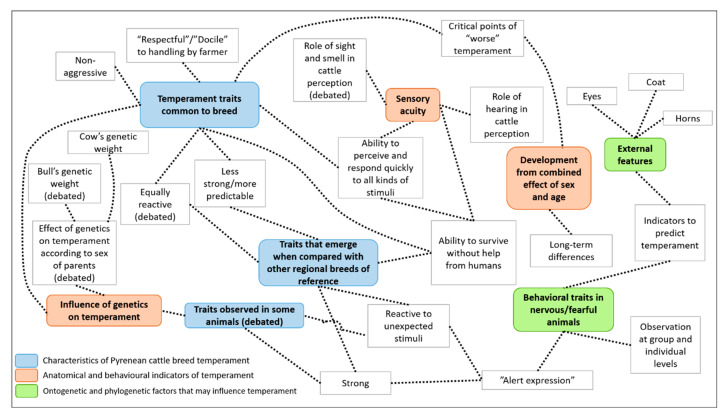
Thematic map of respondents’ perceptions about temperament of Pyrenean cattle in the four focus groups. “Debated” refers to a lack of unanimous agreement within the FGDs regarding that issue.

A thematic analysis was conducted according to the principles described by Braun and Clarke [30], in a process that involved both researchers in the FGDs (moderator and assistant). As a first step, all the FGDs were transcribed verbatim. Each FGD was identified (Huesca FGD 1, Navarra FGD 2, Basque Country FGD 3, Teruel FGD 4), and each participant (P) was assigned a number (P1, P2, etc.). Each of the two researchers read and re-read the whole data set and noted down initial ideas. They then identified each of these ideas with codes (a word or short phrase that symbolically assigns a summative, salient, essence-capturing, and/or evocative attribute for a portion of language data [31]) in a systematic fashion across the entire data set, collating data relevant to each code. Next, both researchers discussed their lists of codes, and condensed the two lists into a single one. Codes were grouped into potential themes to facilitate an interpretative analysis of the data on a broader scale. Both codes and themes were identified first within individual focus groups and then compared across groups. A thematic map was generated to check if the themes worked in relation to the coded extracts and the entire data set (Figure 1). Finally, each theme was named and defined, including all the sub-themes associated with them. The three axes of the discussion proposed at the beginning of the study were used to frame the findings obtained during the FGDs. However, to gain a complete picture of how farmers perceived temperament, all additional information that emerged during the discussions were included in the analysis, going beyond both the guiding questions and the definition of temperament that farmers provided at the beginning of each FGD. When using FGD, agreements or disagreements are fundamental processes that influence the nature and content of responses as the group progresses [26]. Thus, the analysis differentiated, for each of the identified themes, the existence of consensus (pre-existing or built during the discussions) and disagreements. Finally, findings from the thematic analysis were discussed in the light of their coincidences or differences with evidences about livestock temperament coming from behavioural sciences.

## 3. Results

### 3.1. Temperament of Pyrenean Cattle Breed

#### 3.1.1. Definition of Temperament

Each FGD began by asking participants for a definition of temperament. In all FGDs, that definition was related with the general reaction of animals to a wide range of fear-generating stimuli. Examples of such stimuli were human presence, handling, other animals, unusual sounds or objects in specific situations (opening an umbrella). Particularly in FGD 4, several participants also perceived temperament as a stable condition of cattle character, defining it as “personality, the strength or character of the animal; whether it’s aggressive or not, whether it’s calm or agitated by handling” (P1). In the other FGDs, references about the persistent character of temperament focused on attributes that were seen to be problematic for handling: “A cow that escapes from an enclosure will always do that, it’s rude once, it’ll always be rude” (FGD 2, P7). In all FDGs, the description of temperament included multiple adjectives or descriptors, and elicited several discussions focused on which were general characteristics for the breed. There was a shared opinion that although the temperament of Pyrenean cattle can be differentiated from other breeds, within the breed “each cow is unique, in a world of its own” (FGD 3, P4).

#### 3.1.2. Describing the Temperament of Pyrenean Cattle Breed

**Figure 2:**
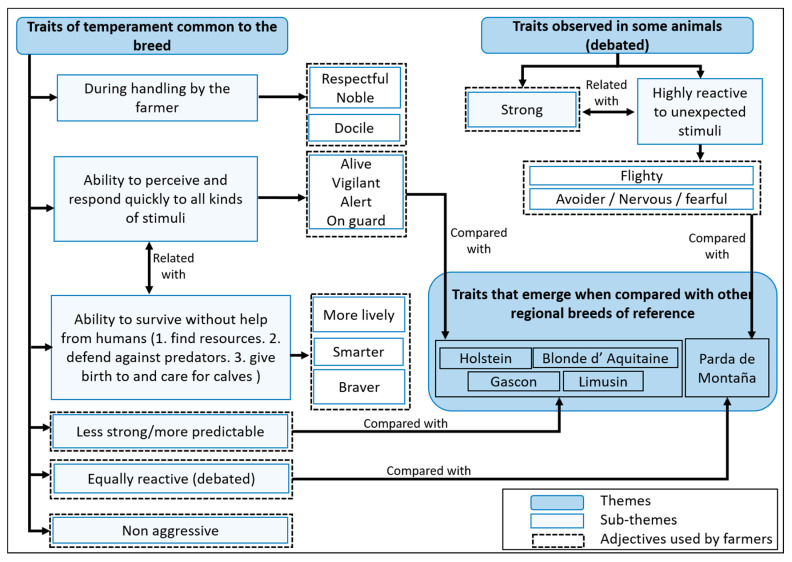
In depth description of Pyrenean cattle temperament according to farmers’ perceptions. “Debated” refers to a lack of unanimous agreement within the FGDs regarding that issue.

When describing the temperament of the Pyrenean cattle, farmers’ discussions focused on temperament traits of the breed and temperament traits observable only in some animals (Figure 2). In terms of the general characteristics of Pyrenean temperament, farmers mentioned adjectives such as “noble” and “docile” to habitual handling carried out by familiar humans and not involving pain. Docility was especially highlighted by participants of FGD 3 and FGD 4, who also defined Pyrenean cattle as very “respectful” of their habitual handler. In their opinion, this would explain apparent dualities in behaviour when interacting with familiar or unfamiliar people. Another consensus (in all FGDs) was that Pyrenean cattle always remain, “attentive”, “alert”, “alive”, “watchful”, or “on guard”. That is, they easily perceive changes in their environment, especially unusual stimuli. Associated with this, participants highlighted the liveliness of the breed, which referred to the animals’ ability to react quickly to negative and positive stimuli that may go unnoticed by other breeds. This capacity was observed by participants in different contexts both on and off the farm, and was considered a positive attribute.

While mentioning the above, participants also highlighted the breed’s ability to survive and adapt to changing environmental conditions. This is especially evident when grazing in open lands without human supervision, where Pyrenean cattle are more adapted (than other breeds) to; (i) find the best pastures, (ii) take advantage of poor pastures, (iii) orient themselves and climb the most rugged mountain areas, (iv) give birth in the mountains, care for calves, and flee or face potential calf predators. In this regard, participants described Pyrenean cattle as “braver”, “livelier”, “fiercer” or “smarter” than other breeds. The above temperaments had also been observed in circumstances other than grazing. For example, a participant of FGD 4 pointed out that the Pyrenean cow “is more aware that I’m going to feed her, or pick up the calves, or whatever. She realizes these things are happening before any other cross-breed or pure breed” (P7).

However, particularly linked to the “alert” adjective, some participants in all FGDs also mentioned the existence of temperament descriptors such as “avoider”, “nervous” or “fearful”. Those terms were used to refer to animals whose flight behaviour is unusually frequent, reactive, fast, and accompanied by a lot of movement. These descriptors were mentioned more frequently in FGD 1, where some participants related them to a general condition: “For me the temperament of this breed, although you can also see it in other breeds, but more in the Pyrenean breed, is that despite being very docile, at a specific moment, due to some action, it reacts in a way that tends to be… that it reacts very quickly and strongly, very savagely” (P3). In this FGD, some participants reported unexpected situations that, in their opinion, caused exaggerated and unjustified flight reactions in their animals. These include, for example, the opening of an umbrella in a stable, a strange sound or a sudden movement by the owner (bending over). The above observations contrast with the other FGDs where participants argued that those temperament descriptors were not generalizable and that the same traits could be found in any breed. One cattle farmer in FGD 4 suggested, as a possible explanation to the sudden or exaggerated cattle movements, that “due to its shape, its makeup, its agility, it appears to make more sudden movements” (P6).

Another descriptor used in the FGDs was “strong temperament”. Some participants described it as a synonym for “nervous”, focusing on the strength of the movements of fearful or avoider animals, while others referred to cattle with “more temperament” that stand up and do not step away from humans: “A farmer friend used to say that the Pyrenean cow had that gaze, and that people looked at it and said, “it seems quite imposing”. No, the Pyrenean cow stares at you that way since it has nothing to be embarrassed about” (FGD 1, P5). In FGD 3 there was a disagreement about whether a “strong” character was a general condition of the breed because some farmers related that term with having “bad manners” (rude disposition). Additionally, there was an agreement in all focus groups that adjectives such as “strong”, “nervous”, “on guard” were not related to aggressiveness. On the contrary, all participants pointed out that aggressiveness was an exceptional condition (1% to 2% of the animals), and that aggressive behaviour tended to be observed in specific situations related with reproduction (the temperament of bulls as a response to females in heat, or peripartum days in cows)**.**

In FGD 1, FGD3, and FGD 4, several discussions arose about the relationship between the breed’s capacity to survive and reproduce without human help (adjectives like “braver”, “livelier”) and those related to the rapid detection and fear reaction even to inconspicuous generating stimuli (adjectives like “alert”, “on guard”, “strong”, “nervous”). Some farmers mentioned that the “strong” temperament of this breed can be an advantage for survival, specifically to protect offspring from predators. Others related an “alert” or “on guard” temperament to a greater probability of survival under extensive mountain grazing conditions. This was related to the opinion that the frequency of handling and artificial selection reduces the aforementioned attributes, although they may improve docility. Other farmers, in contrast, pointed out that it is possible to select for animals that combine both conditions, as nervousness (or fearfulness) is different from the capacity to survive and reproduce without human help. In fact, they suggested that a more nervous temperament could force an animal to remain “hidden in the bushes” and not gain weight.

#### 3.1.3. Comparison with Other Breeds

To place temperament within a broader context, participants were asked to compare Pyrenean cattle to other breeds. Farmers were familiar with Parda de Montaña, Holstein, Limousin, Blonde d’Aquitaine and Gascon breeds. With the exception of Parda de Montaña, allusions to other breeds tended to highlight the positive aspects of the temperament of Pyrenean cattle. Farmers expressed that there is a generalized idea that Parda de Montaña was more docile. However, while FGD 1 were in agreement with this idea, only some participants in the other FGDs were. According to some opinions, the management of the Parda de Montaña is less extensive, and they have more contact with humans, which could explain these differences. Additionally, in all FGDs some farmers brought up their own or third-party experiences related to truck loading or vaccination, in which Parda animals were nervous and even aggressive. These experiences were used to explain that such behaviour may be similar in all breeds in stressful situations, so that the differences between Pyrenean and Parda breeds could just be a matter of reputation or lack of knowledge. Regarding the other breeds, most farmers focused on their negative temperament characteristics. Holstein cattle was suggested to have a “tricky” or “unpredictable” temperament. Limousin and Gascon were described as stronger and more fearful than Pyrenean cattle, as well as Blond d’ Aquitaine. Hence, in all the FGDs, this debate helped to reinforce the idea that Pyrenean cattle had a non-aggressive temperament.

### 3.2. Ontogenetic and Phylogenetic Factors That May Influence Temperament

**Figure 3:**
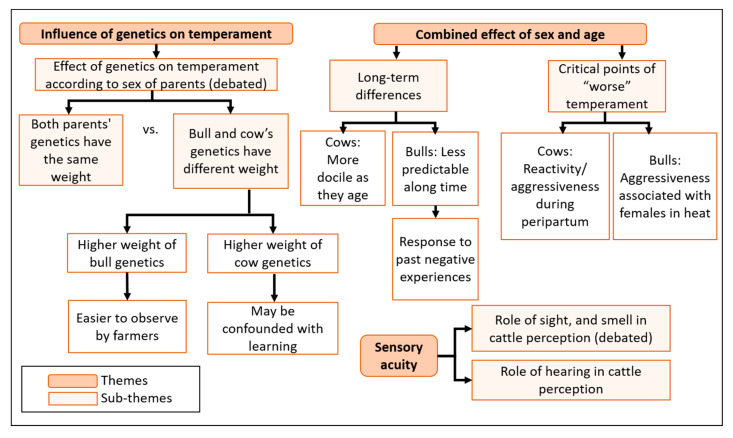
Description of ontogenetic and phylogenetic factors that influence temperament according to farmers’ perceptions. “Debated” refers to a lack of unanimous agreement within the FGDs regarding that issue.

When asked about the role of genetics, age, and sex in temperament, farmers agreed that these factors can make an animal’s temperament different from another. Additionally, sensory acuity of Pyrenean cattle emerged consistently as a new theme in all FGDs, being mentioned as a factor that can influence the way animals perceive stimuli (Figure 3).

#### 3.2.1. Genetics

There was a general consensus that the temperament of parents can be reflected in their offspring. However, there was some disagreement about the degree of this effect, since some participants observed variable effects from the bull or cow parent. Farmers in all FGDs highlighted the heritability of the bull’s temperament in its offspring using explanations like: (i) “if the bull is fearful, the calves are fearful” (FGD 2); (ii) “now we have noticed that, thanks to genetic selection of the bulls, the temperament of the daughters is much better than their mothers” (FGD 4). One participant from FGD 3 suggested a possible explanation for this: “one bull produces many daughters over a short period of time; the bull is the father of all of them and you can relate them to one another, while cows do not have that many daughters” (P5). Most farmers also recognized that cows also affect calf temperament but that it goes beyond genetics, due to the strong cow-calf bond during the first months postpartum. They explained that during this period “if the cow walks, the calf walks, it’s always like that” (FGD 4, P5), while the calf “hardly knows the father” (FGD 3, P7). They explained that the calves observe and learn how their mothers react to stimuli. In the opinion of some participants, that means that the mother can have a greater influence on calf temperament than the bull, especially when calves are born in the mountains and remain isolated with their mother for several months, without human contact.

#### 3.2.2. Age and Sex

Farmers in all FDGs agreed that temperament evolves differently in females and males, with the former being more reactive during their first years of life and becoming increasingly docile with age. As stated by a farmer from FGD 1 “...and then there are some that are bitter their whole lives, but when they turn three they become more grateful. But those first three years, well! I have one that jumped through window years ago, but now follows behind me as calm as can be” (FGD 1, P2). However, they also acknowledged that there can be a few “problematic” animals, whose nervous or reactive temperament is consistent throughout their lifetime. There was some disagreement about when the relatively higher reactivity in cows (two or three years before the first calving) changed towards greater docility, varying from (i) the time of the first calving (two or three years old; FDG 1 and 2); (ii) five or seven years old (FGD 2), and (iii) the third or fourth calving (FGD 3 and 4). The first days after calving were thought to be special risk moments since even the most docile females could become aggressive towards human handlers for 3 to 4 days postpartum, or sometimes even for several weeks.

Regarding breeding bulls, they were thought to be docile during their first year of life and either remain so or become stronger and even aggressive as they aged. FGD 1 commented on both types of cases but, as other FGDs, recognized that their temperament usually became stronger with age. As with cows there as some disagreement about when bulls may become stronger, either starting around age three, or later. One participant explained: “a two or three-year old bull, up until about five years, if he has not done anything to you, normally will remain noble forever. But if after turning three he’s had some ugly behaviour, watch out because one day he’ll go after you. The bull is the most dangerous animal in the herd.” (FGD 4, P7). All FGDs agreed that cows over five years old are usually more docile than bulls, while the latter can become more aggressive in the presence of cows, acknowledging the influence of hormones in bulls. Some farmers in FGD 4 mentioned that breeding bulls may not actually be more aggressive than cows, but it can appear so since they are much bigger. Overall, bulls were also described as “resentful” or “spiteful”, referring to their ability to remember past negative experiences (painful treatment, abuse) and behave aggressively towards specific handlers even weeks or months after the event.

#### 3.2.3. Sensory Acuity

The visual, auditory and olfactory acuity of Pyrenean cattle breed were highlighted in all FGDs: “…and you have to pay attention to their ears, she already knows that I’ve arrived, even though she hasn’t seen me. Or, even if they are all lying down, if there are 20 cows and three look to one side, the other 17 will follow suit and will notice something. And you have not noticed anything, you haven’t seen or heard a thing. And smell, they can smell anything. It’s a truly special cow.” (Navarra). A farmer in FGD 3 also mentioned that cows that cannot see well tend to be more fearful, pointing to a possible relationship between temperament and sensory acuity. All FGDs agreed that the breed has high hearing acuity but there was more disagreement about sight and smell: for FGD 1 and 2 no mention was made of sight while FGD 4 concluded that smell was more important. In FGD 2 there was some debate over this, and no agreement was reached. The following is an example of an exchange in FDG 2 about this topic:

P4. “… I’ve always thought and observed that cows see more through their noses than through their eyes. In the field, for example, they don’t need to have their eyes open, they see you with their nose. Their sense of smell is very well developed.”

P8. “I think that they differentiate between colours … I’ve seen that they can see a field of alfalfa 500 m away and head directly for it.”

P1. “But they smell the grass. In fact, you have a blind animal, and sometimes that happens, … it also gets there.”

P8. “You pass by about 200 m away (from the cows) and a crossing barrier is open, and the cows see it open and head for it. In order to see the barrier, they need to be able to see colour.”

### 3.3. Observation of Temperament Characteristics

**Figure 4:**
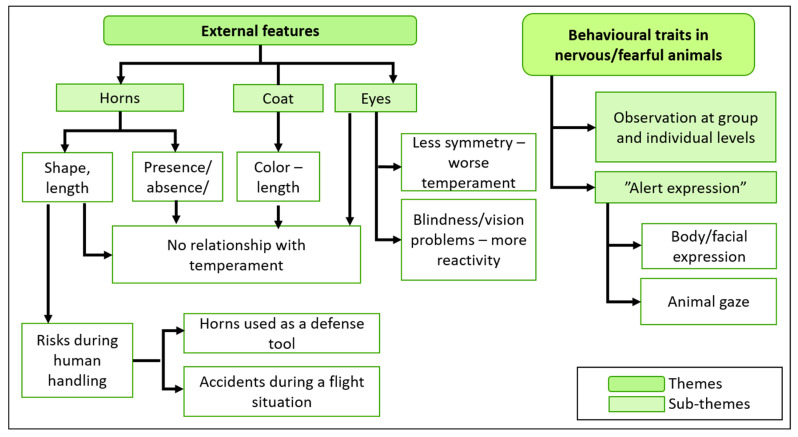
Description of traits observed by farmers for approaching to Pyrenean cattle temperament.

When asked about how to tell the temperament of an animal, farmers discussed about two themes: external features and behaviour (Figure 4).

#### 3.3.1. External Features

There was a general consensus that external features such as conformation and body length are not enough to infer temperament. There was more debate about coat colour, however FGD 3 and 4 farmers agreed that coat colour was not associated with temperament, while in both FGD 1 and 2 there were conflicting opinions (several farmers related the same coat colour with opposite temperament traits). There were a variety of opinions regarding horns and their relationship with temperament, human safety during handling, and social behaviour. Most agreed that the size and shape of horns were not associated with any specific temperament (reaction to fear-generating stimuli) or specific behaviour towards humans. However, FGD 1 and 2 pointed out that the presence of horns represents an additional risk since animals are more likely to harm humans, even if they are not aggressive or especially strong. That can happen when cattle try to escape from a stressful situation. One farmer mentioned that cattle prefer to use horns to defend themselves: “I’ve heard that for a cow with horns, its instinct is to protect itself by hitting you with its horns. It does not kick you. The veterinarians tend to say: a cow with horns doesn’t normally kick, it tries to attack you with its horns…” (FGD 2, P3). In FGD 1 and 2 there were contrasting opinions about whether horns may affect social behaviour. Some farmers expressed that aggressive interactions can be observed between horned and/or dehorned animals, with worse consequences when at least one of them is horned. On the contrary, other farmers (in all FGDs) were convinced that horned animals tended to be more nervous and fight more frequently. Restricted access to food, water, shade or limitations in space, varying widely among farms, were all considered potential triggers for such confrontations. Finally, only one participant from FGD 1 raised a possible hypothesis about the relationship between bulging or non-symmetrical eyes and tougher temperaments, which was neither rejected nor accepted by the rest of the farmers.

#### 3.3.2. Behavioural Features

Farmers in all FGDs expressed that temperament can be elucidated from facial expressions and body position, which are fairly consistent over time and in different situations. Farmers focused on describing the behaviour of the most nervous or fearful animals, which can easily be detected both in the herd and individually. Nervous animals try to hide behind others when confronted with any fear-generating stimuli, and will move away from foreign objects or explore them only after others. When confronted with a stressful situation alone, such as vaccination, nervous animals move a lot or try to run away. Farmers underlined the “alert body/facial expression” as a key indicator of temperament, being easily differentiated from other facial expressions associated with positive (i.e., happiness) and negative (i.e., pain, sadness) emotions. When alert, an animal has its head raised, and ears up and directed forwards (parallel to the ground), with eyes wide open and looking towards the stimulus. Some explained that this body position denotes alertness associated with fear, and indicating a risky situation, preceding the occurrence of flight behaviour with a lot of movement and even aggressive behaviour. Several farmers agreed that the animal’s gaze is crucial to establish when a situation can become risky, but admitted that is very difficult to describe the particular gaze associated with the described expression. As a speaker explained: “You have to be able to tell, when you look at a bull, for example, it doesn’t let you know by its movement but more by the way that he looks at you, there you can see the danger and the same with a cow. But not everyone is able to perceive it, but it’s there, he is warning you already with that gaze” (FGD 1, P1). A possible description of the alert gaze raised by another participant was: “It’s all in the way he looks at you. If he looks at you in a relaxed way, you’ll be ok. If his eyes are bulging or he seems to be scrutinizing you a lot, watch out.” (FGD 2, P4).

## 4. Discussion

Our results help to provide a holistic idea about how farmers perceive and describe the temperament of cattle, in this case, of the Pyrenean breed. In the absence of studies that have previously addressed this issue, this study can represent a starting point for additional research to help understand how farmers approach animal temperament, and how their perceptions affect their handling strategies and their decisions about genetic selection, including the buying, selling, or culling of animals. The definition of temperament provided by farmers in the FGDs is in line with definitions used in farm animal research referring to the consistent response to strange and/or fear-generating stimuli, mainly those associated with humans. Several terms were used that were anthropomorphic, and varied depending on regional differences in language use. However, all the terms seemed related to active and passive reactions to fear [32], such as “strong” or “aggressive” and “nervous” or “fearful”. Some of the terms coincided with those used in studies based on Qualitative Behaviour Assessment, (i.e., “fearful” and “agitated”), where human observers integrate perceived details of behaviour and its context into their judgement of an animal’s overall style of behaviour or ‘behavioural expression’ [33,34]. Farmers even went beyond the description of specific behaviours and defined temperament profiles (e.g., “a nervous cow”), usually based on years of observation. Farmers highlighted individual differences in cattle temperament, but also used terms such as “attentive”, “vigilant” or “alive” to refer to general characteristics of the breed. Both individual and breed-level differences have been recognized as relevant dimensions to analyse temperament and natural history (micro-evolutionary process [35]). Some authors have found individual-level differences within a same breed [36], while others underline the effect of breed on temperament (i.e., [37]) and others have not (i.e., [38]). In any case, other variables such as the production system, human related characteristics and skills, and frequency of handling, can all have an influence on temperament traits beyond breed effects [6,39,40].

When asked further about temperament, a consistent theme that emerged in all FGDs was the ability of Pyrenean cattle to obtain resources, survive and reproduce in extensive mountain grazing conditions, where the availability of resources is changing and sometimes restricted. This was also linked by farmers to the speed of response of these animals to all kinds of stimuli (not only fear-generating ones). Since a large part of the breeding herd is still handled extensively, farmers value these adaptive traits in their cattle, coinciding with preferences of many producers of mountain livestock [41,42]. This could confirm that the behavioral responses that farmers use to describe temperament include traits such as boldness, exploration or activity [1], going beyond those strictly related to human handling. However, the above descriptions are also related to concepts such as robustness [43], adaptability [44,45], rusticity [46], or even maternal behavior [47], which encompasses other dimensions that are different from temperament. This suggests that for farmers, the limits of the concept of temperament could be somewhat lax.

Undesirable temperament is one reason for culling animals [48], a decision that is made by farmers early on (in the first years of animal’s life). However, some temperament traits may not be consistent across stages of ontogeny, from juvenile to adult, especially during major periods of development or times of significant neurological and physiological reorganization [3]. That was observed by farmers who also identified differences in the development of temperament associated with sex: Females tend to become more docile to human handling as they age, coinciding with findings of Tõzsér et al. [49], while in bulls, farmers observed that temperament can be more variable, and early experiences can have a marked influence, which has been also found by Probst et al. [50]. Our findings reinforce the need to incorporate a long-term perspective when analysing the development of temperament, particularly in beef cows and bulls raised under extensive production systems. This is because the development of temperament in breeding cattle has focused mainly on dairy cows [51,52], generally including observation times from a few months to several years. Another important question that warrants further research in beef and in autochthonous breeds revolves around how inheritance interacts with learning (either individual or socially-induced) to determine an animal’s temperament type [53].

Results from all FGDs agree with scientific evidence that asserts that maternal aggressiveness is a critical point for human safety that can be related with other maternal care traits and behavioral reactions to different handling practices [54,55]. Further research on these relations is specially needed in the case of Pyrenean and other autochthonous breeds, as calving and peri-partum can take place under very different conditions, ranging from controlled environments in stables, to mountain landscapes without human contact, where calving cows are exposed to several environmental risks. This means that some defensive maternal behaviours can be more or less useful or convenient according to specific farming conditions, for example, those oriented to protect calves from predators [56]. According to farmer perceptions, external features alone are not reliable enough to judge temperament. For example, the facial hair whorl position, suggested to indicate cattle temperament [11,56,57] was not mentioned in any FGD. The association between coat colour and temperament has been discussed in some beef cattle breeds [49], but there was no consensus in any FGD. A study about Pyrenean cattle published in the 19th century mentions the pure Guipuzcoana breed (an ancestor of the pure Pyrenean breed) had light brown, short and lustrous hair, with a happy and docile temperament [58]. A possible explanation for the above is that farmers preferentially use behavioural traits to interpret temperament, while external features are used to approach other relevant production traits such as weight gain, carcass size or calving difficulty. Therefore, future temperament evaluation protocols involving farmers should prioritize the use of behavioural traits more than external features.

Regarding the presence of horns, farmer opinions about the risks associated with the handling of horned animals coincide with other studies [19] and are in line with general arguments that justify dehorning or disbudding. However, the presence/absence or characteristics of horns were not thought to help predict temperament. There is some evidence that pain associated with disbudding can have an effect on the short-term behavioural responses of animals [59], and that the consequences of this process may be long-lasting [60]. Farmers’ perceptions and available evidence raise the need to study how horned and dehorned cattle react to human handling and other fear-generating stimuli in autochthonous breeds. Farmers separated the relation between horns and social behaviour from temperament, which shares some conceptual ground with behavioural sciences. The role of horns in social dominance and in the monopolization of resources (mates, food, water, resting areas, etc.) is quite clear from behavioural studies [61], so the lack of consensus among farmers about this particular topic may reflect the heterogeneity of effects they observed, which coincides with contrasting evidences of different studies [62]. However, it can also be a consequence of the different types of herds on which the farmers’ opinions are based (only horned, only dehorned, or mixed herds), as their previous experiences limit their options to compare and observe the existing effects [60]. Contrasting opinions on this topic also seem to be mediated by the value that participants assign to horns within their concept of animal’s beauty and of their own self-concept as farmers, aspects common to farmers of different breeds and productive systems [19].

Individual observation of animals has been widely used in animal sciences to describe temperament traits [7,59]. Therefore, it is interesting that to describe temperament, farmers observe the animal both when it is alone, confronting specific stressors, and within the herd. In this latter, farmers are approaching temperament and its relation to the group’s responses. Although there are is some evidence about the link between temperament traits and the social behavior of cattle [7,63], additional research on this issue in beef cattle and especially in autochthonous breeds remains necessary. Conducting studies that incorporate this approach could be relevant for genetic improvement, especially in the small to medium-scale cattle production context, as in several regions worldwide where animals are still being bought on sale yards or at the farm level, where the farmer chooses one or more animals within a group or herd. Therefore, it would be useful for farmers to have tools that allow them to know the temperament traits of an animal in a group context, which are in accordance with their purchase expectations.

The so-called “alert expression” referred to by farmers as a useful indicator of an animal’s temperament, could be considered an outward sign of vigilance. According to Beauchamp [64], vigilance is the state or behaviour of monitoring the surroundings for potential threats. While describing the Pyrenean breed, farmers referred to the two aforementioned views of animal vigilance [64]: On one hand, they highlighted attentiveness or watchfulness as general traits of the breed, which corresponds to the view of vigilance as an internal state that governs how an animal monitors its surroundings. Furthermore, they mentioned the “alert expression”, that relates to how animals actually monitor their surroundings, which may imply that they view vigilance as a behaviour. In the case of nervous or fearful cattle, vigilance seems to be exacerbated. It is also costlier since it can disrupt foraging or other activities [64]. In general, farmers related vigilance with temperament, coinciding with other studies that suggest that dairy cows may alter their vigilance according to their degree of fearfulness toward people and toward different environments [65].

Farmers stressed that “gaze” was associated with the alert expression that indicates fear, and that it can be perceived as a threat to humans. Although they failed to describe it in anatomical terms, watching out for these signs could help to reduce the risks of handling. Other authors have measured the area of white of the eyes as a predictor of temperament [66,67,68], which could be related to the gaze noted by farmers in the present study. The differential use of left or right eyes in stressful situations has also been discussed [69]. However, other facial action units associated with the eye (brow, eye closure, eye orbital, etc.) could be explored further in the Pyrenean breed since they have a role in the expression of other animal emotions [67,70]. Farmers recognized the role of sight, smell, and hearing in vigilance. They also differentiated between the roles that each sense has on the perception of specific stimuli, which has been evidenced in cattle [71,72]. In some FGDs the existence of a relationship between sense acuity and temperament was also raised, although only in terms of nervous animals with vision problems. The question of whether specific behavioural responses of farm animals are due to differences in sensory acuity or temperament traits has been assessed in horses [73], but specific studies in beef cattle are yet needed.

To our knowledge, little has been described about the temperament of this breed. Historically, several documents suggest that it was considered to be quite tame, with animals used for ploughing being kept near people and houses [58,74]. However other authors suggest that animals kept in the mountains for most of the year (with little human contact) are more evasive and can be aggressive [75,76]. The only comparative study of temperament involving Pyrenean cattle found that calves were more susceptible to weaning stress and human presence than Parda de Montaña (Braunvieh or an autochthonous brown cattle) [77]. In the absence of more holistic descriptions about the temperament of the entire breed, our findings provide some additional insights that can work as starting points to deepen our understanding of temperament of this and even of other autochthonous cattle breeds. The disagreements that emerged both within each FGD and between the different FGDs may be influenced by multiple factors, such as the agroecosystem regional conditions, handling and production strategies, local genetic differences of cattle, or the type of human-animal relationships that each farmer has with his animals. As all these factors may vary individually, but the analysis of their influence on the ideas provided in each FGD exceeds the limits of this study. However, the existence of contrasting points of view provides a broad perspective about how farmers approach animal temperament. This is a fundamental step towards the possibility of linking farmers with the assessment of temperament traits for breeding programs, as is now happening within several breed associations in dairy cattle production across the globe [5].

In that sense, the emerging shared views and disagreements encourage the formulation and testing of new hypotheses about, (i) the productive or environmental factors that influence individual or breed level differences in animal temperament, or (ii) the role of human-animal relationships in farmer perceptions about cattle temperament. More broadly, our results serve as a starting point for several future directions for research: (i) The farmers’ perspective about the effect of handling practices and human-animal relationship on cattle temperament. (ii) The farmers’ perceptions about cattle temperament from an individual quantitative approach, which would be useful to verify the consistency between findings from FGD and individual perceptions and to identify the individual/farm level factors that could affect such perceptions. (iii) The role of training on cattle temperament assessment on farmers’ perceptions. (iv) Consistency between farmers’ perceptions about the relationship between temperament and traits such as age, sex, sensory acuity, behavioural response, etc., which should be explored in greater depth using animal-based research. Future analyses that compare breeds based on farmers’ perceptions must take into account that farmers tend to highlight some temperament traits that they consider useful of the breed they manage. Both the results from this study and the above-suggested research topics are relevant to develop training and management strategies aimed at improving animal welfare and, at the same time, increasing farmer safety, and reinforcing the relevance of involving farmers in the description and evaluation of cattle temperament.

## 5. Conclusions

Using FGDs to analyse perceptions about cattle temperament suggests that farmers exhibit a high level of understanding and concern about this topic. They contextualized temperament as a dynamic phenomenon that, although consistent at the individual level, is also subject to changes throughout an individual’s life (ontogeny). They also indicated ontogenetic differences associated with sex, where females tended to become progressively docile, while males were less predictable. An individual’s inherent sensory abilities was thought to play a role in temperament. Farmers did not identify reliable external features that could predict temperament but focused on facial and body expressions, as well as individual behaviours when alone or in a herd context. The reference that farmers make to phenomena such as robustness when describing the temperament of this breed, indicate that the limits they use to delimit the term can be somewhat lax. This should be considered during training programs and when farmers are included in the development of new genetic selection schemes or evaluation protocols.

## Figures and Tables

**Table 1:** Characteristics of participants in the Focus Group Discussions (FGD).

Location	Total	Years of Experience in Pyrenean Cattle Breeding. Freq. (%)			Experience with Other Breeds. Freq. (%)	Include Fattening. Freq.(%)
		3–9	10–20	>20		
FGD 1. Aragon (Province of Huesca)	8	2 (25.0)	4 (50.0)	2 (25.0)	3 (37.5)	1 (12.5)
FGD 2. Navarra	9	3 (33.3)	2 (22.2)	4 (44.4)	6 (66.7)	3 (33.3)
FGD 3. Basque Country	11	2 (18.2)	3 (27.2)	6 (54.5)	6 (54.5)	na.
FGD 4. Aragon (Province of Teruel)	9	2 (22.2)	6 (66.7)	1 (11.1)	6 (66.7)	3 (33.3)

na. = information not available.

**Table 2:** General questions used to prompt discussions of each topic in the FGDs.

Axes of Discussion	Questions
1) Characteristics of Pyrenean cattle breed temperament and comparison with other known breeds	- When you talk about the temperament of cattle, what are you referring to? - How would you describe the temperament of the Pyrenean cattle breed? - What differences have you observed between the temperament of the Pyrenean breed and other breeds reared in this region?
2) Anatomical and behavioural indicators of temperament	- When you observe an animal, what aspects of its appearance allow you to say something about its temperament? - When you observe or interact with an animal, which of its behaviours allow you to say something about its temperament?
3) Ontogenetic and phylogenetic factors that may influence temperament	- When you think back to your cows and bulls, what changes have you detected in their current temperament compared to when they were heifer/steers or calves? - What relationships, if any, have you observed between the temperament of bulls and cows, and that of their offspring?

## Data Availability

Data are not shared due to a privacy protection statement included in the Informed Consent.
